# Supplementary material for: Investigating Mammalian Formins with SMIFH2 Fifteen Years in: Novel Targets and Unexpected Biology
Source: Int J Mol Sci. 2023 May 21;24(10):9058. doi: 10.3390/ijms24109058 (PMC10218792; doi:10.3390/ijms24109058)
Supplement: Supplementary file 1 [file ijms-24-09058-s001.zip › ijms-2360392-supplementary.pdf]

Supplementary Table S1. List of studies employing SMIFH2 with assigned DOI.

Year	Study	DOI	Title
**2022**	Petri et al, 2022	10.1371/journal.pone.0275164	Abnormal left-right organizer and laterality defects in Xenopus embryos after formin inhibitor SMIFH2 treatment
**2022**	Orman et al, 2022	10.1038/s41598-022-17685-z	Alterations to the broad-spectrum formin inhibitor SMIFH2 modulate potency but not specificity
**2022**	Thoidingjam et al, 2022	10.1002/anie.202205231	Small Molecule Inhibitors of Interferon-Induced JAK-STAT Signalling
**2022**	Becker et al, 2022	0.3390/cells11091559	The Cytoskeleton Effectors Rho-Kinase (ROCK) and Mammalian Diaphanous-Related (mDia) Formin Have Dynamic Roles in Tumor Microtube Formation in Invasive Glioblastoma Cells
**2022**	Aloisio et al, 2022	10.1016/j.stemcr.2022.05.002	Arp2/3 complex activity is necessary for mouse ESC differentiation, times formative pluripotency, and enables lineage specification
**2022**	Akwii et al, 2022	10.1007/s10456-022-09831-y	Angiopoietin-2-induced lymphatic endothelial cell migration drives lymphangiogenesis via the β1 integrin-RhoA-formin axis
**2022**	Cangkrama et al, 2022	10.1158/0008-5472.CAN-22-0162	A pro-tumorigenic mDia2-MIRO1 axis controls mitochondrial positioning and function in cancer-associated fibroblasts
**2022**	Chang et al, 2022	10.1016/j.celrep.2022.110390	Substrate rigidity dictates colorectal tumorigenic cell stemness and metastasis via CRAD-dependent mechanotransduction
**2022**	de Poret et al., 2022	10.1111/boc.202100095	Extracellular vesicles containing the I-BAR protein IRSp53 are released from the cell plasma membrane in an Arp2/3 dependent manner
**2022**	Eftekharjoo et al, 2022	10.1021/acsbiomaterials.2c00253	Epithelial Cell-Like Elasticity Modulates Actin-Dependent E-Cadherin Adhesion Organization
**2022**	Fu et al, 2022	10.1242/jcs.258929	Cooperative regulation of adherens junction expansion through epidermal growth factor receptor activation
**2022**	Hori et al, 2022	10.7554/eLife.73542	Microtubule assembly by tau impairs endocytosis and neurotransmission via dynamin sequestration in Alzheimer's disease synapse model
**2022**	Huang et al, 2022	10.1083/jcb.202008116	Cell cortex regulation by the planar cell polarity protein Prickle1
**2022**	Khunta et al, 2022	10.1073/pnas.2204808119	Actin-driven Golgi apparatus dispersal during collective migration of epithelial cells
**2022**	Lång et al, 2022	10.1073/pnas.2201328119	Mechanical coupling of supracellular stress amplification and tissue fluidization during exit from quiescence
**2022**	Lee et al, 2022	10.1016/j.cels.2022.05.005	A molecular clock controls periodically driven cell migration in confined spaces
**2022**	Lin et al, 2022	10.1126/sciadv.abo0323	An AMPK phosphoregulated RhoGEF feedback loop tunes cortical flow–driven amoeboid migration in vivo
**2022**	Liu et al, 2022	10.1002/smll.202104328	Topographic Cues Guiding Cell Polarization via Distinct Cellular Mechanosensing Pathways
**2022**	Logan et al, 2022	10.1242/dev.197442	A Diaphanous and Enabled-dependent asymmetric actin cable array repositions nuclei during Drosophila oogenesis
**2022**	Mishra et al, 2022	10.1242/jcs.259692	Nuclear F-actin and Lamin A antagonistically modulate nuclear shape
**2022**	Mori et al, 2022	10.1016/j.cub.2022.03.057	Extracellular ATP facilitates cell extrusion from epithelial layers mediated by cell competition or apoptosis
**2022**	Romero & Carabeo, 2022	10.1242/jcs.260185	Distinct roles of Chlamydia trachomatis effectors TarP and TmeA in the regulation of formin and Arp2/3 during entry
**2022**	Shi et al, 2022	10.1038/s41467-022-30431-3	Mechanical instability generated by Myosin 19 contributes to mitochondria cristae architecture and OXPHOS

**2022**	Sprenkeler et al, 2022	10.1182/blood.2021013565	Formation of neutrophil extracellular traps requires actin cytoskeleton rearrangements
**2022**	Vanderleest et al, 2022	10.1091/mbc.E21-07-0352	Interface extension is a continuum property suggesting a linkage between AP contractile and DV lengthening processes
**2022**	Yasunaga et al, 2022	10.1038/s41467-022-29741-3	Microridge-like structures anchor motile cilia
**2021**	Adebowale et al, 2021	10.1038/s41563-021-00981-w	Enhanced substrate stress relaxation promotes filopodia-mediated cell migration
**2021**	Ali et al, 2021	10.1080/15592324.2021.1920192	Formins control dynamics of F-actin in the central cell of Arabidopsis thaliana
**2021**	Burrinha et al, 2021	10.1242/jcs.255752	Upregulation of APP endocytosis by neuronal aging drives amyloid-dependent synapse loss
**2021**	Bourdais et al, 2021	10.1242/jcs.259237	Cofilin regulates actin network homeostasis and microvilli length in mouse oocytes
**2021**	Chan et al, 2021	10.1073/pnas.2007526118	Evolutionarily related small viral fusogens hijack distinct but modular actin nucleation pathways to drive cell-cell fusion
**2021**	Chuyen et al, 2021	10.1016/j.devcel.2021.02.026	The Scf/Kit pathway implements self-organized epithelial patterning
**2021**	Eidell et al, 2021	10.1242/jcs.258602	LFA-1 and kindlin-3 enable the collaborative transport of SLP-76 microclusters by myosin and dynein motors
**2021**	Flormann et al, 2021	10.3389/fphy.2021.711860	Oscillatory Microrheology, Creep Compliance and Stress Relaxation of Biological Cells Reveal Strong Correlations as Probed by Atomic Force Microscopy
**2021**	Galotto et al, 2021	10.1093/plphys/kiab435	Myosin XI drives polarized growth by vesicle focusing and local enrichment of F-actin in Physcomitrium patens
**2021**	German et al, 2021	10.1016/j.celrep.2021.109318	Morphological profiling of human T and NK lymphocytes by high-content cell imaging
**2021**	Gaston et al, 2021	10.1038/s41467-021-22482-9	EpCAM promotes endosomal modulation of the cortical RhoA zone for epithelial organization
**2021**	Hiermaier et al, 2021	10.1016/j.jid.2020.09.022	The Actin-Binding Protein α-Adducin Modulates Desmosomal Turnover and Plasticity
**2021**	Hoijman et al, 2021	10.1038/s41586-021-03200-3	Cooperative epithelial phagocytosis enables error correction in the early embryo
**2021**	Huang et al, 2021	10.1038/s41467-020-20355-1	Host CDK-1 and formin mediate microvillar effacement induced by enterohemorrhagic Escherichia coli
**2021**	Ivanov et al, 2021	10.1371/journal.ppat.1010184	Neisseria gonorrhoeae subverts formin-dependent actin polymerization to colonize human macrophages
**2021**	Landino et al, 2021	10.1016/j.cub.2021.10.021	Rho and F-actin self-organize within an artificial cell cortex
**2021**	Le et al, 2021	10.1038/s41467-020-20563-9	Adhesion-mediated heterogeneous actin organization governs apoptotic cell extrusion
**2021**	Liu et al, 2021	10.1002/smll.202104328	Topographic Cues Guiding Cell Polarization via Distinct Cellular Mechanosensing Pathways
**2021**	Kemp et al, 2021	10.1083/jcb.202103074	Micron-scale supramolecular myosin arrays help mediate cytoskeletal assembly at mature adherens junctions
**2021**	Ivanova et al, 2021	10.1126/sciadv.abf3873	Control of synaptic vesicle release probability via VAMP4 targeting to endolysosomes
**2021**	Ma et al, 2021	10.1093/plcell/koab261	Membrane nanodomains modulate formin condensation for actin remodeling in Arabidopsis innate immune responses
**2021**	Kapoor et al, 2021	10.1016/j.celrep.2021.108918	An actomyosin clamp assembled by the Amphiphysin-Rho1-Dia/DAAM-Rok pathway reinforces somatic cell membrane folded around spermatid heads
**2021**	Moore et al, 2021	10.1038/s41586-021-03309-5	Actin cables and comet tails organize mitochondrial networks in mitosis

**2021**	Munjal et al, 2021	10.1016/j.cell.2021.11.025	Extracellular hyaluronate pressure shaped by cellular tethers drives tissue morphogenesis
**2021**	Shi et al, 2021	10.1093/jmcb/mjab070	Actin nucleator formins regulate the tension-buffering function of caveolin-1
**2021**	Majmundar et al, 2021	10.1126/sciadv.abe1386	Recessive NOS1AP variants impair actin remodeling and cause glomerulopathy in humans and mice
**2021**	Prostak et al, 2021	10.1016/j.cub.2021.01.001	The actin networks of chytrid fungi reveal evolutionary loss of cytoskeletal complexity in the fungal kingdom
**2021**	Kohashi et al, 2021	10.1016/j.cub.2021.06.064	Sequential oncogenic mutations influence cell competition
**2021**	Rey-Suarez et al, 2021	10.1091/mbc.E20-10-0685	Actomyosin dynamics modulate microtubule deformation and growth during T-cell activation
**2021**	Lehtimäki et al, 2021	10.7554/eLife.60710	Generation of stress fibers through myosin-driven reorganization of the actin cortex
**2021**	Schmidt et al. 2021	10.1242/jcs.258973	Dia- and Rok-dependent enrichment of capping proteins in a cortical region
**2021**	So-hee et al, 2021	10.11620/IJOB.2021.46.3.99	Mechanisms of tissue factor induction by Porphyromonas gingivalis in human endothelial cells
**2021**	Sakata et al, 2021	10.1111/gtc.12873	Differential effects of the formin inhibitor SMIFH2 on contractility and Ca2+ handling in frog and mouse cardiomyocytes
**2021**	Schiweck et al, 2021	10.1038/s41467-021-21662-x	Drebrin controls scar formation and astrocyte reactivity upon traumatic brain injury by regulating membrane trafficking
**2021**	Sun et al, 2021	10.1038/s41467-021-24375-3	Xanthomonas effector XopR hijacks host actin cytoskeleton via complex coacervation
**2021**	Zhang, et al 2021	10.1093/plphys/kiab085	Analysis of formin functions during cytokinesis using specific inhibitor SMIFH2
**2021**	Xiao et al, 2021	10.1038/s41598-021-95935-2	Integrated computational and experimental pipeline for quantifying local cell–matrix interactions
**2021**	Laplaud et al, 2021	10.1126/sciadv.abe3640	Pinching the cortex of live cells reveals thickness instabilities caused by myosin II motors
**2021**	Fischer et al, 2021	10.1073/pnas.2021135118	Contractility, focal adhesion orientation, and stress fiber orientation drive cancer cell polarity and migration along wavy ECM substrates
**2021**	Ma et al, 2021	10.1016/j.celrep.2021.108884	Formin nanoclustering-mediated actin assembly during plant flagellin and DSF signaling
**2021**	Valencia et al, 2021	10.1016/j.devcel.2021.11.004	Force-dependent activation of actin elongation factor mDia1 protects the cytoskeleton from mechanical damage and promotes stress fiber repair
**2021**	Monzo et al, 2021	10.1016/j.devcel.2021.09.007	Adaptive mechanoproperties mediated by the formin FMN1 characterize glioblastoma fitness for invasion
**2021**	Pocaterra et al. 2021	10.1038/s42003-021-02286-9	Fascin1 empowers YAP mechanotransduction and promotes cholangiocarcinoma development
**2021**	Sugizaki et al, 2021	10.1073/pnas.2019071118	POLArIS, a versatile probe for molecular orientation, revealed actin filaments associated with microtubule asters in early embryos
**2021**	Bischoff et al, 2021	10.1038/s41467-020-20362-2	Filopodia-based contact stimulation of cell migration drives tissue morphogenesis
**2021**	Nishimura, et al 2021	10.1016/j.cdev.2021.203736	Crosstalk between myosin II and formin functions in the regulation of force generation and actomyosin dynamics in stress fibers
**2021**	Nishimura, et al 2021	10.1242/jcs.253708	The Formin Inhibitor, SMIFH2, Inhibits Members of the Myosin Superfamily
**2021**	Tertrais, et al 2021	10.1016/j.ejcb.2021.151161	Phagocytosis is coupled to the formation of phagosome-associated podosomes and a transient disruption of podosomes in human macrophages
**2021**	Vorselem, et al 2021	10.7554/eLife.68627	Phagocytic 'teeth' and myosin-II 'jaw' power target constriction during phagocytosis
**2021**	Yu et al, 2021	10.1083/jcb.202007172	Endophilin A1 drives acute structural plasticity of dendritic spines in response to Ca2+/calmodulin

**2021**	Zhang et al, 2021	10.1007/s12035-021-02531-6	Formin Activity and mDia1 Contribute to Maintain Axon Initial Segment Composition and Structure
**2021**	Zhao et al, 2021	10.1126/sciadv.abg4934	Hydraulic resistance induces cell phenotypic transition in confinement
**2020**	Pfisterer et al, 2020	10.1083/jcb.201906111	FMNL2 regulates dynamics of fascin in filopodia
**2020**	Chan et al, 2020	10.7554/eLife.51358	A viral fusogen hijacks the actin cytoskeleton to drive cell-cell fusion
**2020**	Walpole et al, 2021	10.1016/j.celrep.2020.107721	Inactivation of Rho GTPases by Burkholderia cenocepacia Induces a WASH-Mediated Actin Polymerization that Delays Phagosome Maturation
**2020**	Ma et al, 2020	10.1242/jcs.236794	Arp2/3 nucleates F-actin coating of fusing insulin granules in pancreatic β cells to control insulin secretion
**2020**	Blumenthal et al, 2020	10.7554/eLife.55995	Mouse T cell priming is enhanced by maturation-dependent stiffening of the dendritic cell cortex
**2020**	Cangkrama et al, 2020	10.15252/emmm.201911466	A paracrine activin A–mDia2 axis promotes squamous carcinogenesis via fibroblast reprogramming
**2020**	Colin-York, et al 2020	10.1242/jcs.232322	Distinct actin cytoskeleton behaviour in primary and immortalised T-cells
**2020**	Lionetti et al, 2020	10.1016/j.bpj.2020.04.001	Chromatin and Cytoskeletal Tethering Determine Nuclear Morphology in Progerin-Expressing Cells
**2020**	Cavanaugh et al, 2020	10.1016/j.devcel.2019.12.002	RhoA Mediates Epithelial Cell Shape Changes via Mechanosensitive Endocytosis
**2020**	Davidson et al, 2020	10.15252/embr.201949910	Nesprin-2 accumulates at the front of the nucleus during confined cell migration
**2020**	Moorthy et al, 2020	10.1111/cmi.13196	Matrix stiffness regulates endosomal escape of uropathogenic E. coli
**2020**	Nunes et al, 2020	10.1091/mbc.E20-01-0047	Centrosome–nuclear axis repositioning drives the assembly of a bipolar spindle scaffold to ensure mitotic fidelity
**2020**	Moose et al, 2020	10.1016/j.celrep.2020.02.080	Cancer Cells Resist Mechanical Destruction in Circulation via RhoA/Actomyosin-Dependent Mechano-Adaptation
**2020**	Pinto-Costa et al, 2020	10.1172/JCI125771	Profilin 1 delivery tunes cytoskeletal dynamics toward CNS axon regeneration
**2020**	Pollitt et al, 2020	10.1091/mbc.E20-06-0366	LIM and SH3 protein 1 localizes to the leading edge of protruding lamellipodia and regulates axon development
**2020**	Avarez-Elizondo et al, 2020	10.1007/s10439-020-02679-7	Actin as a Target to Reduce Cell Invasiveness in Initial Stages of Metastasis
**2020**	Kang et al, 2020	10.1091/mbc.E19-11-0602	Angiomotin links ROCK and YAP signaling in mechanosensitive differentiation of neural stem cells
**2020**	Stankevicins et al, 2020	10.1073/pnas.1907845117	Deterministic actin waves as generators of cell polarization cues
**2020**	Chaudhuri, et al 2020	10.1002/adbi.202000143	Modulating T Cell Activation Using Depth Sensing Topographic Cues
**2020**	Limzerwala et al, 2020	10.1038/s43018-020-00116-1	FoxM1 insufficiency hyperactivates Ect2–RhoA–mDia1 signaling to drive cancer
**2020**	Velle et al, 2020	10.1083/jcb.202007158	Conserved actin machinery drives microtubule-independent motility and phagocytosis in Naegleria
**2020**	Ecke et al, 2020	10.1091/mbc.E19-08-0460	Formins specify membrane patterns generated by propagating actin waves
**2020**	Green et al, 2020	10.1111/jth.14735	SMIFH2 inhibition of platelets demonstrates a critical role for formin proteins in platelet cytoskeletal dynamics
**2020**	Nguyen, et al, 2020	10.1007/s12195-019-00603-1	Differential Contributions of Actin and Myosin to the Physical Phenotypes and Invasion of Pancreatic Cancer Cells

**2020**	Liu et al, 2020	10.1096/fj.2019 03033R	Protein diaphanous homolog 1 (Diaph1) promotes myofibroblastic activation of hepatic stellate cells by regulating Rab5a activity and TGF beta receptor endocytosis
**2020**	Cao et al, 2020	10.1038/s41556 -020-0531-y	SPIN90 associates with mDia1 and the Arp2/3 complex to regulate cortical actin organization
**2020**	Kim et al, 2020	10.1038/s41556 -020-0468-1	Optogenetic control of mRNA localization and translation in live cells
**2020**	Thumkeo et al, 2020	10.1126/sciadv. aay2432	mDia1/3-dependent actin polymerization spatiotemporally controls LAT phosphorylation by Zap70 at the immune synapse
**2020**	Teo et al, 2020	10.1016/j.devce l.2020.05.002	Caveolae Control Contractile Tension for Epithelia to Eliminate Tumor Cells
**2020**	Hoffman et al, 2020	10.1091/mbc.E 19-01-0027	Mechanical stress triggers nuclear remodeling and the formation of transmembrane actin nuclear lines with associated nuclear pore complexes
**2020**	Di Cio et al, 2020	10.1016/j.biom aterials.2019.1 19683	Contractile myosin rings and cofilin-mediated actin disassembly orchestrate ECM nanotopography sensing
**2020**	Meyer et al, 2020	10.15252/msb. 20198985	Bile canaliculi remodeling activates YAP via the actin cytoskeleton during liver regeneration
**2020**	Bhosle et al, 2020	10.1038/s41467 -020-17651-1	SLIT2/ROBO1-signaling inhibits macropinocytosis by opposing cortical cytoskeletal remodeling
**2020**	Kühn et al, 2020	10.1016/j.celre p.2020.107638	Actin Assembly around the Shigella-Containing Vacuole Promotes Successful Infection
**2020**	Sil et al, 2020	10.1091/mbc.E 18-11-0715	Dynamic actin-mediated nano-scale clustering of CD44 regulates its meso-scale organization at the plasma membrane
**2020**	Zuidscherw oude et al, 2020	10.1080/095371 04.2020.182252 2	Loss of mDia1 and Fhod1 impacts platelet formation but not platelet function
**2020**	Yaniv et al, 2020	10.1083/jcb.201 903181	Developmental axon regrowth and primary neuron sprouting utilize distinct actin elongation factors
**2020**	Zhang et al, 2020	10.1074/jbc.RA 119.012260	Proline-rich 11 (PRR11) drives F-actin assembly by recruiting the actin-related protein 2/3 complex in human non-small cell lung carcinoma
**2020**	Zhu et al, 2020	10.1126/scienc e.abd270	Developmental clock and mechanism of de novo polarization of the mouse embryo
**2019**	Meka et al, 2019	10.15252/embr. 201947743	Radial somatic F-actin organization affects growth cone dynamics during early neuronal development
**2019**	Ajeti et al, 2019	10.1038/s41567 -019-0485-9	Wound healing coordinates actin architectures to regulate mechanical work
**2019**	Aspenstrom, 2019	10.3390/cells80 70759	The Intrinsic GDP/GTP Exchange Activities of Cdc42 and Rac1 Are Critical Determinants for Their Specific Effects on Mobilization of the Actin Filament System
**2019**	Corkins et al, 2019	10.1371/journa l.pone.0221698	Divergent roles of the Wnt/PCP Formin Daam1 in renal ciliogenesis
**2019**	Kalappurak kal et al, 2019	10.1016/j.cell.2 019.04.037	Integrin Mechano-chemical Signaling Generates Plasma Membrane Nanodomains that Promote Cell Spreading
**2019**	Miller and Blystone, 2019	10.1002/jcb.286 94	The carboxy-terminus of the formin FMNL1ɣ bundles actin to potentiate adenocarcinoma migration
**2019**	Ditlev et al, 2019	10.7554/eLife.4 2695	A composition-dependent molecular clutch between T cell signaling condensates and actin
**2019**	Dudin et al, 2019	10.7554/eLife.4 9801	A unicellular relative of animals generates a layer of polarized cells by actomyosin-dependent cellularization
**2019**	Eaton et al, 2019	10.1091/mbc.E 19-02-0115	Expansion and contraction of the umbrella cell apical junctional ring in response to bladder filling and voiding

**2019**	Gao et al, 2019	10.1073/pnas.1903763116	Genetic dissection of active forgetting in labile and consolidated memories in Drosophila
**2019**	Luo et al, 2019	10.1038/s41467-019-10241-w	Atypical function of a centrosomal module in WNT signalling drives contextual cancer cell motility
**2019**	Roesler et al, 2019	10.3389/fncel.2019.00330	Myosin XVI Regulates Actin Cytoskeleton Dynamics in Dendritic Spines of Purkinje Cells and Affects Presynaptic Organization
**2019**	Jaumouillé, et al, 2019	10.1038/s41556-019-0414-2	Coupling of β2 integrins to actin by a mechanosensitive molecular clutch drives complement receptor-mediated phagocytosis
**2019**	Hsieh et al, 2019	10.1038/s41467-019-08924-5	Omegasome-proximal PtdIns(4,5)P2 couples F-actin mediated mitoaggregate disassembly with autophagosome formation during mitophagy
**2019**	Jalal et al, 2019	10.1242/jcs.220780	Actin cytoskeleton self-organization in single epithelial cells and fibroblasts under isotropic confinement
**2019**	Lu et al, 2019	10.1073/pnas.1910166116	Membrane curvature underlies actin reorganization in response to nanoscale surface topography
**2019**	Melzer et al, 2019	10.3390/ijms20040876	Involvement of Actin Cytoskeletal Components in Breast Cancer Cell Fusion with Human Mesenchymal Stroma/Stem-Like Cells
**2019**	Strieder-Barboza et al, 2019	10.1038/s41598-019-56242-z	Advanced glycation end-products regulate extracellular matrix-adipocyte metabolic crosstalk in diabetes.
**2019**	Mohan et al, 2019	10.1016/j.devcel.2019.04.007	Enhanced Dendritic Actin Network Formation in Extended Lamellipodia Drives Proliferation in Growth-Challenged Rac1P29S Melanoma Cells
**2019**	Puleo et al, 2019	10.1083/jcb.201902101	Mechanosensing during directed cell migration requires dynamic actin polymerization at focal adhesions
**2019**	Xia et al, 2019	10.1016/j.celrep.2019.06.089	Nanoscale Architecture of the Cortical Actin Cytoskeleton in Embryonic Stem Cells
**2019**	Stephenson et al, 2019	10.1016/j.devcel.2019.01.016	Rho Flares Repair Local Tight Junction Leaks
**2019**	Kita et al, 2019	10.1091/mbc.E19-02-0126	Spindle–F-actin interactions in mitotic spindles in an intact vertebrate epithelium
**2019**	Plessner et al, 2019	10.1016/j.isci.2019.04.022	Centrosomal Actin Assembly Is Required for Proper Mitotic Spindle Formation and Chromosome Congression
**2019**	Lee et al, 2019	10.1128/MCB.00107-19	CCN1–Yes-Associated Protein Feedback Loop Regulates Physiological and Pathological Angiogenesis
**2019**	Guetta-Terrier et al, 2019	10.1083/jcb.201501106	Protrusive waves guide 3D cell migration along nanofibers
**2019**	Chistensen, et al 2019	doi: 10.1091/mbc.e19-08-0463	Chlamydomonas reinhardtii formin FOR1 and profilin PRF1 are optimized for acute rapid actin filament assembly
**2019**	Vohnoutka et al, 2019	10.1091/mbc.E19-08-0442	The focal adhesion scaffold protein Hic-5 regulates vimentin organization in fibroblast
**2019**	Zhang et al, 2019	10.1016/j.bbrc.2019.09.047	The migration direction of hair cell nuclei is closely related to the perinuclear actin filaments in Arabidopsis
**2019**	Alieva et al, 2019	10.1038/s41467-019-10964-w	Myosin IIA and formin dependent mechanosensitivity of filopodia adhesion
**2019**	Gulvady et al, 2019	10.1091/mbc.E18-10-0629	Hic-5 regulates Src-induced invadopodia rosette formation and organization
**2019**	Glebov, 2019	10.1002/cbin.11226	Distinct molecular mechanisms control levels of synaptic F-actin
**2019**	Khalilgharibi, et al 2019	10.1038/s41567-019-0516-6	Stress relaxation in epithelial monolayers is controlled by the actomyosin cortex
**2019**	Eftekharjoo et al, 2019	10.1016/j.bbrc.2019.01.035	Fibrillar force generation by fibroblasts depends on formin

**2019**	Moreau et al, 2019	10.1016/j.devcel.2019.03.024	Macropinocytosis Overcomes Directional Bias in Dendritic Cells Due to Hydraulic Resistance and Facilitates Space Exploration
**2019**	Shamipour et al, 2019	10.1016/j.cell.2019.04.030	Bulk Actin Dynamics Drive Phase Segregation in Zebrafish Oocytes
**2019**	van Bommel, et al 2019	10.15252/embj.2018101183	F-actin patches associated with glutamatergic synapses control positioning of dendritic lysosomes
**2019**	Roper et al, 2019	10.7554/eLife.48093	B cells extract antigens at Arp2/3-generated actin foci interspersed with linear filaments
**2019**	Singh et al, 2019	10.1128/mBio.02434-19	Measles Virus Ribonucleoprotein Complexes Rapidly Spread across Well-Differentiated Primary Human Airway Epithelial Cells along F-Actin Rings
**2019**	Pettee et al., 2019	10.3390/cancers11030392	Targeting the mDia Formin-Assembled Cytoskeleton Is an Effective Anti-Invasion Strategy in Adult High-Grade Glioma Patient-Derived Neurospheres
**2019**	Tsopoulidis et al, 2019	10.1126/sciimmunol.aav1987	T cell receptor–triggered nuclear actin network formation drives CD4+ T cell effector functions
**2019**	Yan et al, 2019	10.1016/j.isci.2019.05.040	In Vivo F-Actin Filament Organization during Lymphocyte Transendothelial and Interstitial Migration Revealed by Intravital Microscopy
**2019**	Sun et al, 2019	10.1016/j.celrep.2019.10.128	Maintenance of Primary Hepatocyte Functions In Vitro by Inhibiting Mechanical Tension-Induced YAP Activation
**2019**	Ong et al, 2019	10.1242/jcs.224378	Multiple feedback mechanisms fine-tune Rho signaling to regulate morphogenetic outcomes
**2019**	Huang et al, 2019	10.3390/cells8101264	Arp2/3-Branched Actin Maintains an Active Pool of GTP-RhoA and Controls RhoA Abundance
**2019**	Drewry et al, 2019	10.1038/s41564-019-0504-8	The secreted kinase ROP17 promotes Toxoplasma gondii dissemination by hijacking monocyte tissue migration
**2019**	Inoue et al, 2019	10.15252/embj.201899630	Actin filaments regulate microtubule growth at the centrosome
**2019**	Molinie et al, 2019	10.1038/s41422-019-0160-9	Cortical branched actin determines cell cycle progression
**2019**	Vogt et al, 2019	10.1038/s41467-019-10171-7	Anchoring cortical granules in the cortex ensures trafficking to the plasma membrane for post-fertilization exocytosis
**2019**	Yanakieva et al, 2019	10.1083/jcb.201901077	Cell and tissue morphology determine actin-dependent nuclear migration mechanisms in neuroepithelia
**2019**	Bucki et al, 2019	10.1074/jbc.RA118.005552	Lateral distribution of phosphatidylinositol 4,5-bisphosphate in membranes regulates formin- and ARP2/3-mediated actin nucleation
**2018**	Argenzio et al, 2018	10.1074/jbc.RA118.002779	Profilin binding couples chloride intracellular channel protein CLIC4 to RhoA–mDia2 signaling and filopodium formation
**2018**	Brigida et al, 2018	10.1182/blood-2018-07-863431	T-cell defects in patients with ARPC1B germline mutations account for combined immunodeficiency
**2018**	Bun et al, 2018	10.7554/eLife.31469	A disassembly-driven mechanism explains F-actin-mediated chromosome transport in starfish oocytes
**2018**	Gao et al, 2018	10.1002/jcp.27078	Cirrhotic stiffness affects the migration of hepatocellular carcinoma cells and induces sorafenib resistance through YAP
**2018**	Carisey et al, 2018	10.1016/j.cub.2017.12.044	Nanoscale Dynamism of Actin Enables Secretory Function in Cytolytic Cells
**2018**	Sepúlveda-Ramírez et al, 2018	10.1016/j.ydbio.2018.03.015	Cdc42 controls primary mesenchyme cell morphogenesis in the sea urchin embryo
**2018**	Velle et al, 2018	10.1371/journal.ppat.1007485	Enteropathogenic E. coli relies on collaboration between the formin mDia1 and the Arp2/3 complex for actin pedestal biogenesis and maintenance

**2018**	Liu et al, 2018	10.1016/j.molp.2018.09.004	Actin Polymerization Mediated by AtFH5 Directs the Polarity Establishment and Vesicle Trafficking for Pollen Germination in Arabidopsis
**2018**	Huang et al, 2018	10.1038/s41467-018-04710-x	Rice actin binding protein RMD controls crown root angle in response to external phosphate
**2018**	Aihara et al, 2018	10.1242/jcs.216317	Cell injury triggers actin polymerization to initiate epithelial restitution
**2018**	Chakrabarty et al, 2018	10.1083/jcb.201711022	Processive flow by biased polymerization mediates the slow axonal transport of actin
**2018**	Dvorak et al, 2018	10.1371/journal.pone.0195278	Carcinoma associated fibroblasts (CAFs) promote breast cancer motility by suppressing mammalian Diaphanous-related formin-2 (mDia2)
**2018**	Fernández-Barrera et al, 2018	10.1083/jcb.201702157	The actin-MRTF-SRF transcriptional circuit controls tubulin acetylation via α-TAT1 gene expression
**2018**	Ford et al, 2018	10.1371/journal.ppat.1007051	Chlamydia exploits filopodial capture and a macropinocytosis-like pathway for host cell entry
**2018**	Ray et al, 2018	10.1073/pnas.1808052115	Intrinsic cellular chirality regulates left–right symmetry breaking during cardiac looping
**2018**	Higuchi-Sanabria et al, 2018	10.1091/mbc.E18-06-0362	Spatial regulation of the actin cytoskeleton by HSF-1 during aging
**2018**	Weise-Cross et al, 2018	10.1152/ajpheart.00664.2017	Actin polymerization contributes to enhanced pulmonary vasoconstrictor reactivity after chronic hypoxia
**2018**	Logue et al, 2018	10.1038/s41388-017-0071-5	c-Src activity is differentially required by cancer cell motility modes
**2018**	Kim et al, 2018	10.1096/fj.201801429RR	Stress hormone signaling through β-adrenergic receptors regulates macrophage mechanotype and function
**2018**	Kruppa et al, 2018	10.1016/j.devcel.2018.01.007	Myosin VI-Dependent Actin Cages Encapsulate Parkin-Positive Damaged Mitochondria
**2018**	Kudryashova et al, 2018	10.1016/j.cub.2018.03.065	Actin Cross-Linking Toxin Is a Universal Inhibitor of Tandem-Organized and Oligomeric G-Actin Binding Proteins
**2018**	Yasuda-Yamahara et al, 2018	10.1016/j.matbio.2018.01.003	FERMT2 links cortical actin structures, plasma membrane tension and focal adhesion function to stabilize podocyte morphology
**2018**	Mahuzier et al, 2018	10.1038/s41467-018-04676-w	Ependymal cilia beating induces an actin network to protect centrioles against shear stress
**2018**	Rana et al, 2018	10.1091/mbc.E17-05-0325	Formin-dependent TGF-beta signaling for epithelial to mesenchymal transition
**2018**	Silveira et al, 2018	10.1189/jlb.3A0916-388RR	TNF induces neutrophil adhesion via formin-dependent cytoskeletal reorganization and activation of beta-integrin function
**2018**	Fessendon et al, 2018	10.1083/jcb.201703145	Dia1-dependent adhesions are required by epithelial tissues to initiate invasion
**2018**	Pfanzelter et al, 2018	10.1083/jcb.201708091	Septins suppress the release of vaccinia virus from infected cells
**2018**	Schrank et al, 2018	10.1038/s41586-018-0237-5	Nuclear ARP2/3 drives DNA break clustering for homology-directed repair
**2018**	McRae et al, 2018	10.1080/21688370.2017.1405774	Characterization of cell-cell junction changes associated with the formation of a strong endothelial barrier
**2018**	Prashar et al, 2018	10.3389/fcimb.2018.00133	Small Rho GTPases and the Effector VipA Mediate the Invasion of Epithelial Cells by Filamentous Legionella pneumophila
**2018**	Ripoli et al, 2018	10.1083/jcb.201709055	Myosin VI and branched actin filaments mediate membrane constriction and fission of melanosomal tubule carriers
**2018**	Terry et al, 2018	10.1073/pnas.1722281115	Capping protein regulates actin dynamics during cytokinetic midbody maturation

**2018**	Copeland et al, 2018	10.1091/mbc.E18-02-0088	Actin-dependent regulation of cilia length by the inverted formin FHDC1
**2018**	Xiang et al, 2018	10.4049/jimmunol.1701805	β1 Integrins Are Required To Mediate NK Cell Killing of Cryptococcus neoformans
**2018**	Schell et al, 2018	10.1016/j.devcel.2018.11.011	ARP3 Controls the Podocyte Architecture at the Kidney Filtration Barrier
**2018**	Tabdanov, et al, 2018	10.1038/s41467-018-07290-y	Bimodal sensing of guidance cues in mechanically distinct microenvironments
**2018**	Sakamoto, et al 2018	10.1371/journal.pbio.2004874	mDia1/3 generate cortical F-actin meshwork in Sertoli cells that is continuous with contractile F-actin bundles and indispensable for spermatogenesis and male fertility
**2018**	Sathe et al, 2018	10.1038/s41467-018-03955-w	Small GTPases and BAR domain proteins regulate branched actin polymerisation for clathrin and dynamin-independent endocytosis
**2018**	Matsuzawa et al, 2018	10.1016/j.celrep.2018.05.070	α-Catenin Controls the Anisotropy of Force Distribution at Cell-Cell Junctions during Collective Cell Migration
**2018**	Zenker et al, 2018	10.1016/j.cell.2018.02.035	Expanding Actin Rings Zipper the Mouse Embryo for Blastocyst Formation
**2018**	Fenix et al, 2018	10.7554/eLife.42144	Muscle-specific stress fibers give rise to sarcomeres in cardiomyocytes
**2018**	Hirata et al, 2018	10.1111/cas.13816	Active K-RAS induces the coherent rotation of epithelial cells: A model for collective cell invasion in vitro
**2018**	Whitson et al, 2018	10.1038/nm.4476	Noncanonical hedgehog pathway activation through SRF–MKL1 promotes drug resistance in basal cell carcinomas
**2018**	Williams & Kay, 2018	10.1242/jcs.213736	The physiological regulation of macropinocytosis during Dictyostelium growth and development
**2018**	Wu et al, 2018	10.1083/jcb.201802039	Actin and microtubule cross talk mediates persistent polarized growth
**2017**	Bharadwaj et al, 2017	10.1038/ncomms14348	αV-class integrins exert dual roles on α5β1 integrins to strengthen adhesion to fibronectin
**2017**	Beach et al, 2017	10.1038/ncb3463	Actin dynamics and competition for myosin monomer govern the sequential amplification of myosin filaments
**2017**	Chew et al, 2017	10.1083/jcb.201701104	Actin turnover maintains actin filament homeostasis during cytokinetic ring contraction
**2017**	Collins et al, 2017	10.1073/pnas.1618676114	Changes in E-cadherin rigidity sensing regulate cell adhesion
**2017**	Delaney, et al 2017	10.1073/pnas.1700247114	Distinct functions of diaphanous-related formins regulate HIV-1 uncoating and transport
**2017**	Foster et al, 2017	10.1101/gad.304501.117	Mutual dependence of the MRTF–SRF and YAP–TEAD pathways in cancer-associated fibroblasts is indirect and mediated by cytoskeletal dynamics
**2017**	Fritzsche et al, 2017	10.1126/sciadv.1603032	Cytoskeletal actin dynamics shape a ramifying actin network underpinning immunological synapse formation
**2017**	Schwartz et al, 2017	10.1038/s41598-017-01324-z	Lamins and nesprin-1 mediate inside-out mechanical coupling in muscle cell precursors through FHOD1
**2017**	Hu et al, 2017	10.1038/ncb3466	Long-range self-organization of cytoskeletal myosin II filament stacks
**2017**	Hui et al, 2017	10.1073/pnas.1614291114	Dynamic microtubules regulate cellular contractility during T-cell activatio
**2017**	Lopes et al, 2017	10.1083/jcb.201608094	Membrane nanoclusters of FcγRI segregate from inhibitory SIRPα upon activation of human macrophages
**2017**	Qu et al, 2017	10.1016/j.molp.2017.05.002	Organizational Innovation of Apical Actin Filaments Drives Rapid Pollen Tube Growth and Turning
**2017**	Qu et al, 2017	10.1083/jcb.201701045	Stabilization of dynamic microtubules by mDia1 drives Tau-dependent Aβ1–42 synaptotoxicity
**2017**	Spadaro et al, 2017	10.1016/j.cub.2017.11.014	Tension-Dependent Stretching Activates ZO-1 to Control the Junctional Localization of Its Interactors

**2017**	Wu et al, 2017	10.1016/j.bpj.2016.12.035	Two Distinct Actin Networks Mediate Traction Oscillations to Confer Focal Adhesion Mechanosensing
**2017**	Miller et al, 2017	10.1242/jcs.195099v	Non-canonical activity of the podosomal formin FMNL1γ supports immune cell migration
**2017**	Nager et al, 2017	10.1016/j.cell.2016.11.036	An Actin Network Dispatches Ciliary GPCRs into Extracellular Vesicles to Modulate Signaling
**2017**	Oakes et al., 2017	10.1038/ncomms15817	Optogenetic control of RhoA reveals zyxin-mediated elasticity of stress fibres
**2017**	Öztürk et al, 2017	10.1016/j.yexcr.2017.08.033	RhoA activation and nuclearization marks loss of chondrocyte phenotype in crosstalk with Wnt pathway
**2017**	Panzica et al, 2017	10.1083/jcb.201702020	F-actin prevents interaction between sperm DNA and the oocyte meiotic spindle in C. elegans
**2017**	Parisis et al, 2017	10.15252/embj.201796585	Initiation of DNA replication requires actin dynamics and formin activity
**2017**	Pfisterer et al, 2017	10.1038/ncomms14858	Role for formin-like 1-dependent acto-myosin assembly in lipid droplet dynamics and lipid storage
**2017**	Archarya et al, 2017	10.1016/j.celrep.2017.02.078	Mammalian Diaphanous 1 Mediates a Pathway for E-cadherin to Stabilize Epithelial Barriers through Junctional Contractility
**2017**	Taniguchi et al, 2017	10.1083/jcb.201704085	An apicosome initiates self-organizing morphogenesis of human pluripotent stem cells
**2017**	Tomoshige et al, 2017	10.1016/j.ygcen.2017.08.021	Cytoskeleton-related regulation of primary cilia shortening mediated by melanin-concentrating hormone receptor 1
**2017**	Balasanyan et al, 2017	10.1016/j.celrep.2017.11.046	Structure and Function of an Actin-Based Filter in the Proximal Axon
**2017**	Soykan et al, 2017	10.1016/j.neuron.2017.02.011	Synaptic Vesicle Endocytosis Occurs on Multiple Timescales and Is Mediated by Formin-Dependent Actin Assembly
**2017**	Stromeyer et al, 2017	10.1038/nmat5023	Fibronectin-bound $\alpha5\beta1$ integrins sense load and signal to reinforce adhesion in less than a second
**2017**	van der Kammen et al, 2017	10.1242/dev.156323	Knockout of the Arp2/3 complex in epidermis causes a psoriasis-like disease hallmarked by hyperactivation of transcription factor Nrf2
**2017**	Wyse et al, 2017	10.1016/j.bbrc.2017.01.087	mDia2 and CXCL12/CXCR4 chemokine signaling intersect to drive tumor cell amoeboid morphological transitions
**2017**	You et al, 2017	10.1158/0008-5472.CAN-17-0657	SPIN90 Depletion and Microtubule Acetylation Mediate Stromal Fibroblast Activation in Breast Cancer Progression
**2017**	Zaritsky et al, 2017	10.1083/jcb.201609095	Diverse roles of guanine nucleotide exchange factors in regulating collective cell migration
**2017**	Zhang et al, 2017	10.1093/jmicro/dfx015	In vivo dynamics of the cortical actin network revealed by fast-scanning atomic force microscopy
**2016**	Acharya et al, 2016	10.1242/jcs.173674	KIF17 regulates RhoA-dependent actin remodeling at epithelial cell–cell adhesions
**2016**	Bernau et al, 2016	10.1165/rcmb.2016-0104OC	Tensin 1 Is Essential for Myofibroblast Differentiation and Extracellular Matrix Formation
**2016**	Dubey et al, 2016	10.1016/j.devcel.2016.07.001	Localized, Reactive F-Actin Dynamics Prevents Abnormal Somatic Cell Penetration by Mature Spermatids
**2016**	Jasnin et al, 2016	10.1016/j.str.2016.05.004	Actin Organization in Cells Responding to a Perforated Surface, Revealed by Live Imaging and Cryo-Electron Tomography
**2016**	Nejedla et al, 2016	10.1091/mbc.E15-11-0799	Profilin connects actin assembly with microtubule dynamics
**2016**	Farina et al, 2016	10.1038/ncb3285	The centrosome is an actin-organizing centre
**2016**	Luo et al, 2016	10.1371/journal.pone.0163915	Formin DAAM1 Organizes Actin Filaments in the Cytoplasmic Nodal Actin Network
**2016**	Toshima et al, 2016	10.7554/eLife.10276	Yeast Eps15-like endocytic protein Pan1p regulates the interaction between endocytic vesicles, endosomes and the actin cytoskeleton

**2016**	Heuserman n et al, 2016	10.1083/jcb.201 506084	Exosomes surf on filopodia to enter cells at endocytic hot spots, traffic within endosomes, and are targeted to the ER
**2016**	Gargini et al, 2016	10.1016/j.celre p.2016.10.064	WIP Drives Tumor Progression through YAP/TAZ-Dependent Autonomous Cell Growth
**2016**	Delevoye et al, 2016	10.1016/j.cub.2 015.11.020	BLOC-1 Brings Together the Actin and Microtubule Cytoskeletons to Generate Recycling Endosomes
**2016**	Panzer et al, 2016	10.1242/jcs.177 691	The formins FHOD1 and INF2 regulate inter- and intra-structural contractility of podosomes
**2016**	Ziske et al, 2016	10.1016/j.bbrc. 2016.02.049	SMIFH2-mediated mDia formin functional inhibition potentiates chemotherapeutic targeting of human ovarian cancer spheroids
**2016**	Cao et al, 2016	10.1104/pp.15. 01321	Profilin-Dependent Nucleation and Assembly of Actin Filaments Controls Cell Elongation in Arabidopsis
**2016**	Michael et al, 2016	10.1016/j.devce l.2016.03.008	Coronin 1B Reorganizes the Architecture of F-Actin Networks for Contractility at Steady-State and Apoptotic Adherens Junctions
**2016**	Kuroda et al, 2016	10.1038/srep34 809	Diaphanous gene mutation affects spiral cleavage and chirality in snails
**2016**	Leithner et al, 2016	10.1038/ncb342 6	Diversified actin protrusions promote environmental exploration but are dispensable for locomotion of leukocytes
**2016**	Borinskaya et al, 2016	10.1091/mbc.E 14-11-1555	Integration of linear and dendritic actin nucleation in Nck-induced actin comets
**2016**	Basu et al, 2016	10.1523/ENEU RO.0302-16.2016	Arp2/3 and VASP Are Essential for Fear Memory Formation in Lateral Amygdala
**2016**	Fritzsche et al., 2016	10.1126/sciadv. 1501337	Actin kinetics shapes cortical network structure and mechanics
**2016**	Rengarajan et al, 2016	10.1371/journa l.ppat.1005603	Endothelial Cells Use a Formin-Dependent Phagocytosis-Like Process to Internalize the Bacterium Listeria monocytogenes
**2016**	Rao et al, 2016	10.1091/mbc.E 16-06-0429	Formin-mediated actin polymerization at cell–cell junctions stabilizes E-cadherin and maintains monolayer integrity during wound repair
**2016**	Monzo et al, 2016	10.1091/mbc.E 15-08-0565	Mechanical confinement triggers glioma linear migration dependent on formin FHOD3
**2016**	Moore et al, 2016	10.1038/ncom ms12886	Dynamic actin cycling through mitochondrial subpopulations locally regulates the fission–fusion balance within mitochondrial networks
**2016**	Murugesan et al, 2016	10.1083/jcb.201 603080	Formin-generated actomyosin arcs propel T cell receptor microcluster movement at the immune synapse
**2016**	Sari-Hassoun et al, 2016	10.1016/j.bcp.2 015.12.013	Cucurbitacin I elicits the formation of actin/phospho-myosin II co-aggregates by stimulation of the RhoA/ROCK pathway and inhibition of LIM-kinase
**2016**	Sedzinski et al, 2016	10.1016/j.devce l.2015.12.013	Emergence of an Apical Epithelial Cell Surface In Vivo
**2016**	Stritt et al, 2016	10.1182/blood-2015-10-675629	A gain-of-function variant in DIAPH1 causes dominant macrothrombocytopenia and hearing loss
**2016**	Hadjiantoni ou et al, 2016	10.1098/rsif.20 16.0613	Physical confinement signals regulate the organization of stem cells in three dimensions
**2016**	Qu et al, 2016	10.1091/mbc.e 16-10-0727	Periodic actin structures in neuronal axons are required to maintain microtubules
**2016**	Wales et al, 2016	10.7554/eLife.1 9850	Calcium-mediated actin reset (CaAR) mediates acute cell adaptations
**2016**	Wen et al, 2016	10.1038/ncom ms12604	Actin dynamics provides membrane tension to merge fusing vesicles into the plasma membrane
**2015**	Eghiaian et al, 2015	10.1016/j.bpj.2 015.01.016	Structural, Mechanical, and Dynamical Variability of the Actin Cortex in Living Cells
**2015**	Grega-Larson et al, 2015	10.1091/mbc.E 15-06-0443	Cordon bleu promotes the assembly of brush border microvilli

**2015**	Roy et al, 2015	10.1083/jcb.201506110	A dynamic formin-dependent deep F-actin network in axons
**2015**	Heisler et al, 2015	10.1126/science.aab4090	ACD toxin–produced actin oligomers poison formin-controlled actin polymerization
**2015**	Vargas et al, 2015	10.1038/ncb3284	Innate control of actin nucleation determines two distinct migration behaviours in dendritic cells
**2015**	Barry et al, 2015	10.1083/jcb.201501081	Open source software for quantification of cell migration, protrusions, and fluorescence intensities
**2015**	Vitriol et al, 2015	10.1016/j.celrep.2015.03.033	Two Functionally Distinct Sources of Actin Monomers Supply the Leading Edge of Lamellipodia
**2015**	Henson et al, 2015	10.1091/mbc.E14-07-1244	Arp2/3 complex inhibition radically alters lamellipodial actin architecture, suspended cell shape, and the cell spreading process
**2015**	Katsuno et al, 2015	10.1016/j.celrep.2015.06.048	Actin Migration Driven by Directional Assembly and Disassembly of Membrane-Anchored Actin Filaments
**2015**	Lee et al, 2015	10.1016/j.cels.2015.07.001	Functional Hierarchy of Redundant Actin Assembly Factors Revealed by Fine-Grained Registration of Intrinsic Image Fluctuations
**2015**	Kanellos et al, 2015	10.1016/j.celrep.2015.10.056	ADF and Cofilin1 Control Actin Stress Fibers, Nuclear Integrity, and Cell Survival
**2015**	Taniguchi et al, 2015	10.1016/j.stemcr.2015.10.015	Lumen Formation Is an Intrinsic Property of Isolated Human Pluripotent Stem Cells
**2015**	Makhija et al, 2015	10.1073/pnas.1513189113	Nuclear deformability and telomere dynamics are regulated by cell geometric constraints
**2015**	Tornavaca et al, 2015	10.1083/jcb.201404140	ZO-1 controls endothelial adherens junctions, cell–cell tension, angiogenesis, and barrier formation
**2015**	Fattouh et al, 2015	10.1093/infdis/jiu546	The Diaphanous-Related Formins Promote Protrusion Formation and Cell-to-Cell Spread of Listeria monocytogenes
**2015**	Paul et al, 2015	10.1083/jcb.201502040	$\alpha 5\beta 1$ integrin recycling promotes Arp2/3-independent cancer cell invasion via the formin FHOD3
**2015**	Suraneni et al, 2015	10.1091/mbc.E14-07-1250	A mechanism of leading-edge protrusion in the absence of Arp2/3 complex
**2015**	Rotty et al, 2015	10.1016/j.devcel.2014.10.026	Profilin-1 Serves as a Gatekeeper for Actin Assembly by Arp2/3-Dependent and -Independent Pathways
**2015**	Phng et al, 2015	10.1016/j.devcel.2014.11.017	Formin-Mediated Actin Polymerization at Endothelial Junctions Is Required for Vessel Lumen Formation and Stabilization
**2015**	Arden et al, 2015	10.1091/mbc.E14-11-1502	Small-molecule agonists of mammalian Diaphanous-related (mDia) formins reveal an effective glioblastoma anti-invasion strategy
**2015**	Erfemov et al, 2015	10.1016/j.bbamcr.2015.05.008	Distinct impact of targeted actin cytoskeleton reorganization on mechanical properties of normal and malignant cells
**2015**	Kim et al, 2015	10.1371/journal.pone.0123438	Small molecule inhibitor of formin homology 2 domains (SMIFH2) reveals the roles of the formin family of proteins in spindle assembly and asymmetric division in mouse oocytes.
**2015**	Isogai et al, 2015	10.1038/srep09802	SMIFH2 has effects on Formins and p53 that perturb the cell cytoskeleton.
**2015**	Ramanathan et al, 2015	10.1038/ncb3098	Cdk1-dependent mitotic enrichment of cortical myosin II promotes cell rounding against confinement
**2015**	Tabdanov, et al, 2015	10.1039/c5ib00032g	Micropatterning of TCR and LFA-1 ligands reveals complementary effects on cytoskeleton mechanics in T cells
**2015**	Tee et al, 2015	10.1038/ncb3137	Cellular chirality arising from the self-organization of the actin cytoskeleton
**2015**	Saha et al, 2015	10.1091/mbc.E15-06-0397	Diffusion of GPI-anchored proteins is influenced by the activity of dynamic cortical actin
**2015**	Wakayama et al, 2015	10.1016/j.devcel.2014.11.024	Cdc42 Mediates Bmp-Induced Sprouting Angiogenesis through Fmnl3-Driven Assembly of Endothelial Filopodia in Zebrafish
**2015**	Sorce et al, 2015	10.1038/ncomms9872	Mitotic cells contract actomyosin cortex and generate pressure to round against or escape epithelial confinement

**2014**	Wong et al, 2014	10.1073/pnas.1412285111	Fibroblasts probe substrate rigidity with filopodia extensions before occupying an area
**2014**	Avasthi et al, 2014	10.1016/j.cub.2014.07.038	Actin Is Required for IFT Regulation in Chlamydomonas reinhardtii
**2014**	Guet et al, 2014	10.1016/j.cub.2014.06.048	Mechanical Role of Actin Dynamics in the Rheology of the Golgi Complex and in Golgi-Associated Trafficking Events
**2014**	Lechuga et al., 2014	10.1091/mbc.e14-03-0815	Loss of γ-cytoplasmic actin triggers myofibroblast transition of human epithelial cells
**2014**	Kajita et al., 2014	10.1038/ncomms5428	Filamin acts as a key regulator in epithelial defence against transformed cells
**2014**	Harris et al., 2014	10.1242/jcs.142349	Formation of adherens junctions leads to the emergence of a tissue-level tension in epithelial monolayers
**2014**	Beckham et al., 2014	10.1371/journal.pone.0100943	Arp2/3 Inhibition Induces Amoeboid-Like Protrusions in MCF10A Epithelial Cells by Reduced Cytoskeletal-Membrane Coupling and Focal Adhesion Assembly
**2014**	Tien and Chang, 2014	10.1038/onc.2013.241	
**2014**	Pettee et al., 2014	10.1371/journal.pone.0090371	An mDia2/ROCK Signaling Axis Regulates Invasive Egress from Epithelial Ovarian Cancer Spheroids
**2014**	Jennings et al., 2014	10.1182/blood-2014-02-557843	RhoA determines disease progression by controlling neutrophil motility and restricting hyperresponsiveness
**2013**	Nedeva et al, 2013	10.1038/ncomms3165	Synthetic polyamines promote rapid lamellipodial growth by regulating actin dynamics
**2013**	Ucar et al, 2013	10.1242/jcs.130476	The Mos–MAPK pathway regulates Diaphanous-related formin activity to drive cleavage furrow closure during polar body extrusion in starfish oocytes
**2013**	Buvall et al., 2013		
**2013**	Murk et al., 2013	10.1242/jcs.125146	The antagonistic modulation of Arp2/3 activity by N-WASP, WAVE2 and PICK1 defines dynamic changes in astrocyte morphology
**2013**	Luo et al., 2013	10.1083/jcb.201210123	Analysis of the local organization and dynamics of cellular actin networks
**2013**	Iskratsch et al., 2013	10.1016/j.devcel.2013.11.003	FHOD1 Is Needed for Directed Forces and Adhesion Maturation during Cell Spreading and Migration
**2013**	Yu et al., 2013		
**2013**	Aragona et al., 2013	10.1016/j.cell.2013.07.042	A Mechanical Checkpoint Controls Multicellular Growth through YAP/TAZ Regulation by Actin-Processing Factors
**2013**	Rao et al., 2013		
**2013**	Wilson et al., 2013	10.1038/ncomms3896	Mechanisms of leading edge protrusion in interstitial migration
**2013**	Goldspink et al., 2013	10.1242/jcs.129759	The microtubule end-binding protein EB2 is a central regulator of microtubule reorganisation in apico-basal epithelial differentiation
**2013**	Fritzsche et al., 2013	10.1091/mbc.e12-06-0485	Analysis of turnover dynamics of the submembranous actin cortex
**2013**	Sandbo et al., 2013		
**2012**	Rosero et al., 2012	10.1093/jxb/ers351	AtFH1 formin mutation affects actin filament and microtubule dynamics in Arabidopsis thaliana
**2012**	Chin et al., 2012	10.1371/journal.pone.0046949	Actin Recruitment to the Chlamydia Inclusion Is Spatiotemporally Regulated by a Mechanism That Requires Host and Bacterial Factors
**2012**	Miklavc et al., 2012	10.1242/jcs.105262	Actin coating and compression of fused secretory vesicles are essential for surfactant secretion – a role for Rho, formins and myosin II

**2012**	Oakes et al., 2012		
**2012**	Wyse et al., 2012	10.1371/journal.pone.0045085	Dia-Interacting Protein (DIP) Imposes Migratory Plasticity in mDia2-Dependent Tumor Cells in Three-Dimensional Matrices
**2012**	Tang and Brieher, 2012	10.1083/jcb.201103116	α-Actinin-4/FSGS1 is required for Arp2/3-dependent actin assembly at the adherens junction
**2011**	Li et al., 2011		
**2011**	Poincloux et al., 2011	10.1073/pnas.1010396108	Contractility of the cell rear drives invasion of breast tumor cells in 3D Matrigel
**2009**	Rizvi et al., 2009		

Supplementary Table S2. Formin proteins on which SMIFH2 has been shown to inhibit the actin polymerization activity. Official protein names for human formins are indicated, whereas aliases are used for the mouse orthologues (mDia1 = Diap1; mDia2 = Diap3). Note that FH1-FH2-C-term and FH1-FH2 were assayed in [29] and in [28], respectively.

Formin	IC_{50} [Reference]
**DIAPH1/mDia1**	30 ± 10 [29]/~10 [28]
**DIAPH2**	6 ± 3 [29]
**DIAPH3/mDia2**	~10 [28]
**INF2**	10 ± 5 1 [29]
**FMNL3**	27 0 ± 1 [29]
